# Identification of two telomere-proximal fission yeast DNA replication origins constrained by nearby cis-acting sequences to replicate in late S phase

**DOI:** 10.12688/f1000research.1-58.v1

**Published:** 2012-12-04

**Authors:** Amna Chaudari, Joel A Huberman

**Affiliations:** 1 Department of Molecular and Cellular Biology, Roswell Park Cancer Institute, Buffalo, NY, 14263, USA

## Abstract

Telomeres of the fission yeast, *Schizosaccharomyces pombe*, are known to replicate in late S phase, but the reasons for this late replication are not fully understood. We have identified two closely-spaced DNA replication origins, 5.5 to 8 kb upstream from the telomere itself. These are the most telomere-proximal of all the replication origins in the fission yeast genome. When located by themselves in circular plasmids, these origins fired in early S phase, but if flanking sequences closer to the telomere were included in the circular plasmid, then replication was restrained to late S phase – except in cells lacking the replication-checkpoint kinase, Cds1. We conclude that checkpoint-dependent late replication of telomere-associated sequences is dependent on nearby cis-acting sequences, not on proximity to the physical end of a linear chromosome.

## Introduction

The genome of the fission yeast, *Schizosaccharomyces pombe*, is organized into three chromosomes with a total of six protein-DNA telomere structures. The outermost portions of the telomeres consist of telomerase-encoded simple-sequence repeats rich in G residues as read in the 5´ to 3´ direction toward the end of the chromosome. Fission yeast telomeric simple-sequence repeats appear, at first sight, to be more complicated than those of other organisms ^1^, but analysis of motif frequencies ^2^ and analysis of the sequence of the fission yeast telomerase template RNA ^3^ show that the simple-sequence repeats consist mainly of the heptamer GGTTACA with variable numbers of additional G residues (due to polymerase-template slippage) and variable extents of template-coded spacer sequences.

**Figure 1.**
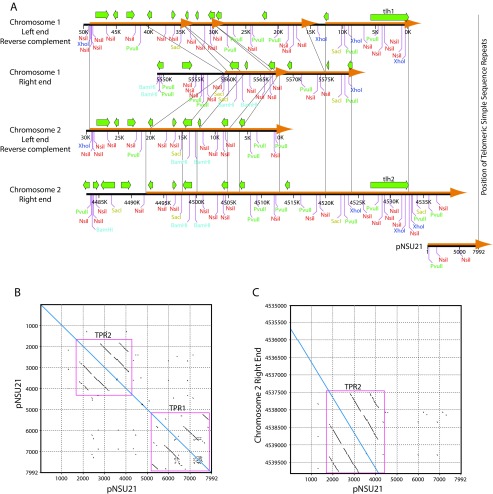
Comparison of telomere-associated sequences (TAS) at the ends of fission yeast chromosomes 1 and 2. ( **A**) The October, 2008, versions ( ftp://ftp.sanger.ac.uk//pub2/yeast/pombe/Chromosome_contigs/OLD/20080922) of the nucleotide sequences from the ends of fission yeast chromosomes 1 and 2 were aligned with each other on the basis of restriction map similarities and Pustell DNA matrix alignments using MacVector software ( http://www.macvector.com/). The left ends of chromosomes 1 and 2 were reverse-complemented so that their sequences would have the same polarity as those of the right ends of chromosomes 1 and 2. The green arrows show the locations and orientations of documented genes (dubious and pseudo genes were omitted). The orange arrows show regions of high sequence similarity between the four chromosome ends. The positions of deletions and an insertion within these highly similar regions in the ends of chromosome 1 (compared to the ends of chromosome 2) are shown by additional orange arrowheads (deletions) or a gap in the orange arrow (insertion). Thin black guide lines are also provided, to facilitate the alignments of similar sequences despite the presence of deletions and an insertion. The far right end of chromosome 2 overlaps for about 4 kb (panel **C**) with the left end of pNSU21, a clone of a 7992-bp telomeric *Hin*dIII fragment prepared by Neal Sugawara ^1^. Here we have used the reverse-complement of the pNSU21 nucleotide sequence, as determined partially by Neal Sugawara ^1^ and completely by the Sanger Centre fission yeast sequencing project ( ftp://ftp.sanger.ac.uk//pub2/yeast/sequences/pombe/telomeres). We have employed the reverse complement (and we’ve changed the numbering correspondingly) so that (i) the pNSU21 sequence would have the same orientation as the right end of chromosome 2, and (ii) so that the G-rich strand of the simple-sequence repeats would be the top strand, read from 5´ to 3´. In this numbering system, the simple-sequence repeats are at nucleotides 7881–7992. The *Hin*dIII site at position 1 of pNSU21 is not shown in the diagram. The only restriction sites shown are *Bam*HI, *Nsi*I, *Pvu*II, *Sac*I, and *Xho*I. These have been given distinct colors to enhance the reader’s ability to perceive similarities between restriction site patterns. A thin vertical line at the right of the diagram shows the estimated position of the simple-sequence repeats for all four chromosome ends. In other words, this line provides an estimate of how far the nucleotide sequence of each chromosome end would extend if the sequence were established all the way to the telomere. ( **B**) A Pustell DNA matrix diagram was prepared (using MacVector software) of pNSU21 against itself, in order to identify significant internal repeat sequences. The conditions employed were: window size 20, minimum % score 100, hash value 6, jump 1. Two families of direct internal repeats were identified. These are called "Telomere-Proximal Repeats" (TPR). The TPRs closest to the telomere are called TPR1, while those further from the telomere are called TPR2. The line of sequence identity is colored blue. ( **C**) A Pustell DNA matrix diagram was prepared of the right end of chromosome 2 against pNSU21, using the same conditions as in ( **B**). The blue line shows the region of (near) sequence identity. Approximately 3840 nucleotides would need to be added to the sequence of the right end of chromosome 2 for it to reach the simple-sequence repeats. The TPR2 family of internal direct repeats is included in the sequence of the right end of chromosome 2.

At the ends of most chromosomes, telomere-associated sequences (TAS) are located internal to the simple-sequence repeats ^1^. In all fission yeast strains, TAS are found at both ends of the two larger chromosomes (1 and 2). Chromosome 3 contains arrays of ribosomal DNA (rDNA) repeats near both ends. In some strains, these rDNA repeats directly abut the simple-sequence repeats ^1^, while in other strains there are TAS between the rDNA and the simple-sequence repeats at one or both chromosome 3 ends ^1, 4, 5^. The complete TAS array consists of approximately 50 kb, forming large inverted repeats at the ends of chromosomes 1 and 2 ( Figure 1A). Most of this sequence is "unique" (except for the fact that each cell contains 4–6 copies of TAS), but there are clusters of direct repeats within the first 3 kb and also 4 to 6 kb away from the telomere ( Figure 1B, C). Within the reference *Schizosaccharomyces pombe* genome sequence of October, 2008 ( ftp://ftp.sanger.ac.uk//pub2/yeast/pombe/Chromosome_contigs/OLD/20080922), only the right end of chromosome 2 extended sufficiently close to the telomere to include any of these direct repeats. The ends of the other chromosomes were less completely sequenced. Fortunately, the right end of chromosome 2 provides an excellent model for the structure of the other chromosome ends ( Figure 1A).

Moser *et al.* ^6, 7^ have shown that the replication forks emanating from the most telomere-proximal origins in fission yeast reach telomeres asymmetrically, with the leading strand polymerase arriving about 20 minutes ahead of the lagging strand polymerase. The lagging strand delay appears to provide an opportunity for differential processing of the leading and lagging strands at the telomere. What could be the mechanism of this telomere-specific replication fork asymmetry? There may be something special about the replication origins located closest to telomeres, or *cis*-acting sequences that accelerate leading strand synthesis and/or decelerate lagging strand synthesis (perhaps in collaboration with bound proteins) may be located between telomere-proximal origins and the telomeres themselves. In either case, it would be of great interest to identify and characterize the telomere-proximal replication origins in fission yeast.

Kim and Huberman ^8^ previously used two-dimensional (2D) agarose gel electrophoresis to study the origin function and replication timing in fission yeast of the TAS-derived telomere-proximal *Hin*dIII restriction fragments (bounded by a *Hin*dIII restriction site on one side and a telomere on the other side; 7–8 kb each). They found that these fragments were replicated in late S phase. They also observed that these fragments usually produced only Y-arc signals, indicative of passive replication by forks from a centromere-proximal origin or by forks from an internal origin close to one of their ends. Occasionally, however, bubble-arc signals indicative of active origin firing within the middle portions of the fragments were also observed. These results suggested that the replication origins closest to telomeres in fission yeast may be located within these *Hin*dIII fragments. In this paper, we describe our characterization of two replication origins located in the centromere-proximal 30% of the *Hin*dIII fragments. These two late-firing origins appear to be the closest origins to the telomeres.

Late telomere replication has also been observed in the budding yeast, *Saccharomyces cerevisiae* ^9– 12^. In budding yeast cells treated with hydroxyurea (HU), lateness of telomere replication is maintained by the Rad53-dependent replication checkpoint ^13^.

In this paper, we report our investigation of the causes of late telomere replication timing in fission yeast. In agreement with previous studies ^8, 14– 16^, we found that the replication check-point regulates telomere replication timing in fission yeast as in budding yeast. In addition, we found that late replication appears to be a consequence of *cis*-acting sequences located between the two telomere-proximal replication origins and the telomere itself.

## Results

### Localization of ARS elements within a Telomeric *Hin*dIII Fragment (THF)

**Figure 2.**
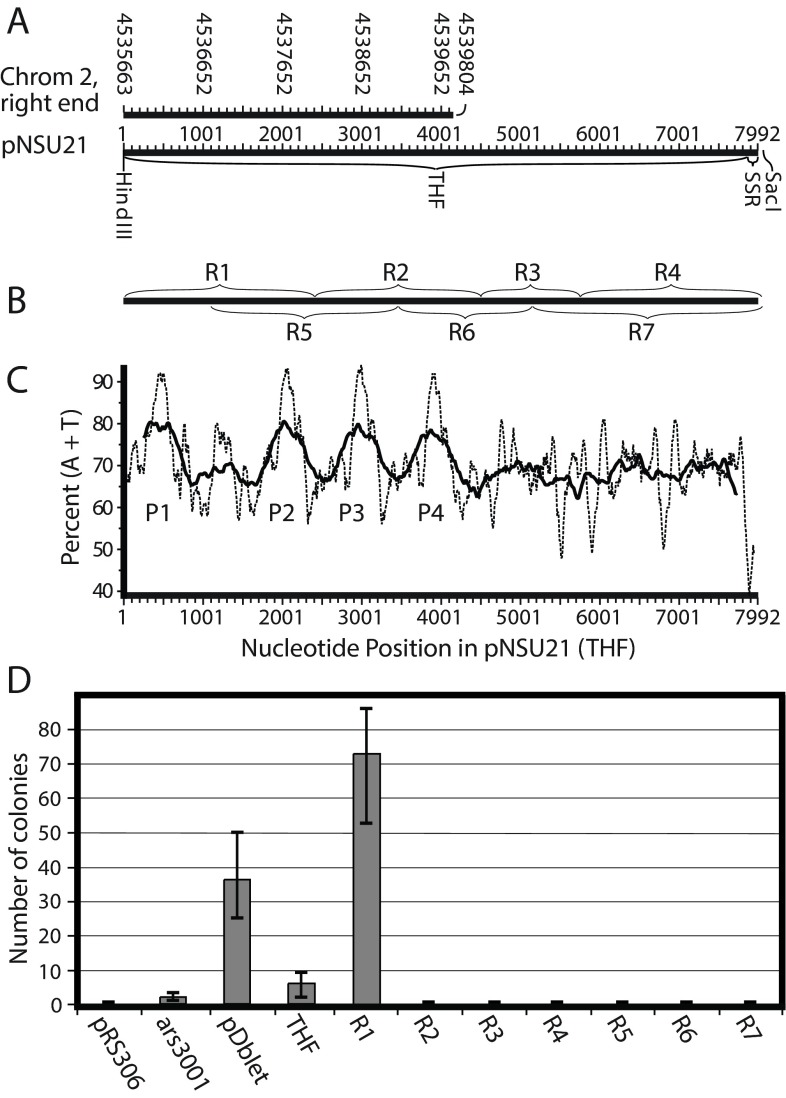
Identification of two ARS elements in the 8-kb Telomeric *Hin*dIII Fragment (THF). ( **A**) Diagram showing overlap between the right end of Chromosome 2 and the THF, which was cloned by Neal Sugawara ^1^ as pNSU21. ( **B**) Relative positions within the THF of the smaller regions (R1–R7) generated by PCR. ( **C**) AT content in sliding windows of 100 bp (dotted line) and 500 bp (solid line) along the THF. P1–P4 are peaks of unusually high AT content. ( **D**) Results of ARS assays for plasmids containing the sequences in panel B or controls. The results are the averages ± standard deviation of 3 experiments, with triplicate plating in each experiment.

Previous observations ^8^ suggested that the telomere-proximal *Hin*dIII restriction fragments of fission yeast were likely to contain replication origins. These would necessarily be the most telomere-proximal origins in the genome. Sugawara ^1^ cloned five such fragments, all of similar sizes and with similar restriction maps. Sugawara cloned these five fragments between the *Hin*dIII and *Sac*I sites in the plasmid, pMLC12. The nucleotide sequences of the inserts were subsequently determined by the fission yeast genome sequencing project and are available from the Sanger Centre ( ftp://ftp.sanger.ac.uk//pub2/yeast/sequences/pombe/telomeres). Of these sequences, that of pNSU21 (7992 bp) most closely resembles the sequence of the right end of chromosome 2 in the region of overlap ( Figure 1, Figure 2A), so we chose pNSU21 for further study.

The classic method for identifying potential yeast DNA replication origins is to test the abilities of stretches of the DNA of interest to serve as replication origins in a plasmid context. Stretches of DNA that meet this criterion are called "Autonomously Replicating Sequences" or ARS elements ^17^. To identify potential origins within pNSU21, we extracted the ~8-kb telomere *Hin*dIII- *Sac*I fragment (hereafter called THF for Telomeric *Hin*dIII Fragment) from pMLC12 and re-cloned it into the yeast shuttle vector, pRS306 ^18^. We also used PCR to create smaller subfragments of the THF, called R1–R7 (for Region 1 through Region 7), and we cloned them into pRS306 ( Figure 2B; Table S1).

**Table S1.** 

Region & Orientation ^a^	Positions in reference sequence ^b^	Primer sequence ^c^
Region 1 Forward	2062–2086 pRS306-RC	CCTCGAGGTCGACGGTATCGATAAG ^d^
Region 1 Reverse	2385–2403 RC	C CGG **GATCC**CGCGACTCATGCACACTACATCC
Region 2 Forward	2384–2407	CG **GAATTC**CGGGGATGTAGTGTGCATGAGTGAAT
Region 2 Reverse	4489–4508 RC	CG **GGATCC**CGGTGAACCATAACACACTCAC
Region 3 Forward	4489–4508	CG **GAATTC**CGGTGAGTGTGTTATGGTTCAC
Region 3 Reverse	5737–5763 RC	CG **GGATCC**CGCGCTGCACAACTGTAATCTACTTAATC
Region 4 Forward	5747–5766	GC **TCTAGA**GCATTACAGTTGTGCAGCGTAG
Region 4 Reverse	1925–1949 pRS306	TTTCCCAGTCACGACGTTGTAAAAC ^e^
Region 5 Forward	1091–1113	CG **GAATTC**CGGTGAGTAGAGAGAGTAGAGTAAG
Region 5 Reverse	3462–3487 RC	CG **GGATCC**CGTTTACTGCTCGTTACCCACCTGAATC
Region 6 Forward	3462–3487	CG **GAATTC**CGGATTCAGGTGGGTAACGAGCAGTAAA
Region 6 Reverse	5129–5159 RC	CG **GGATCC**CGCAACCCAACTTCATTTTCTTCATTCATTTTC
Region 7 Forward	5123–5148	CG **GGATCC**CGGGTAATGAAAATGAATGAAGAAAATG
Region 7 Reverse	1925–1949 pRS306	TTTCCCAGTCACGACGTTGTAAAAC ^e^

^a^The relative positions of the regions are indicated in Figure 1B.

^b^Unless otherwise indicated, the reference sequence is the reverse complement of pNSU21, which may be downloaded from the Sanger Institute web site ( ftp://ftp.sanger.ac.uk/pub/yeast/sequences/pombe/telomeres/). We used the reverse complement of pNSU21 so that the orientation of pNSU21 would match that of the right end of chromosome 2 and so that the "top" strand of the sequence would contain the G-rich portion of the telomere simple-sequence repeats. The reference sequence for the vector, pRS306, is the sequence with GenBank accession number U03438. Those cases in which a primer sequence corresponds to the "bottom" strand (in other words, the reverse complement of the top strand) of the reference sequence are indicated by "RC" for "reverse complement".

^c^Where indicated, the following restriction sites with flanking non-coded nucleotides were appended to the 5´ ends of the template-encoded primers. The flanking non-coded nucleotides are indicated by plain type, while the restriction sites themselves are indicated by bold face:

*Bam*HI: CG **GGATCC**CG.

*Eco*RI: CG **GAATTC**CG.

*Xba*I: GC **TCTAGA**GC.

Additional non-coded nucleotides, indicated by violet coloring (CG), were inserted where indicated in the region 1 reverse primer.

^d^The region 1 forward primer was located in the pRS306 vector, and the resulting PCR fragment contained a *Hin*dIII site at its left end.

^e^The region 4 reverse and region 7 reverse primers were located in the pRS306 vector, and the resulting PCR fragments contained a *Sac*I site at the right end.

When designing the positions of the boundaries of subfragments R1–R7, we took into account information derived from the nucleotide sequence of the THF regarding the likely positions of replication origins. Because the N-terminal portion of fission yeast Orc4 contains nine "AT-hook" motifs, fission yeast ORC binds preferentially to AT-rich sequences ^19– 21^. Indeed, the locations of functional replication origins in fission yeast can be predicted with surprising accuracy based on AT content alone ^22, 23^. Figure 2C displays graphs of the AT content of the THF in sliding windows of 100 bp (thin line) and 500 bp (thick line). In both graphs, four peaks (P1–P4) of high AT content are apparent.

To test whether any of these AT-rich peaks (or other regions in the THF) might serve as replication origins, we used the transformation frequency assay, which is based on counting the number of colonies formed when yeast cells are transformed by a plasmid containing the sequence to be tested for origin activity. The transformed cells are able to form colonies at high frequency only if the plasmid they have been transformed with contains DNA that can serve as an origin of replication for the plasmid. We used pRS306 without an insert as a negative control ( Figure 2D). As positive controls, we employed a weak origin, *ars3001* ^24, 25^ and the strong compound origin in the plasmid, pDblet ^26^. The intact THF had weak activity, similar to that of *ars3001* ( Figure 2D). In contrast, subfragment R1 was more active than pDblet, while R2–R7 were completely inactive ( Figure 2D). These results show that one or more plasmid replication origins (ARS elements) are contained within R1 and suggest that there may be *cis*-acting sequences outside of R1 that can suppress origin activity.

**Figure 3.**
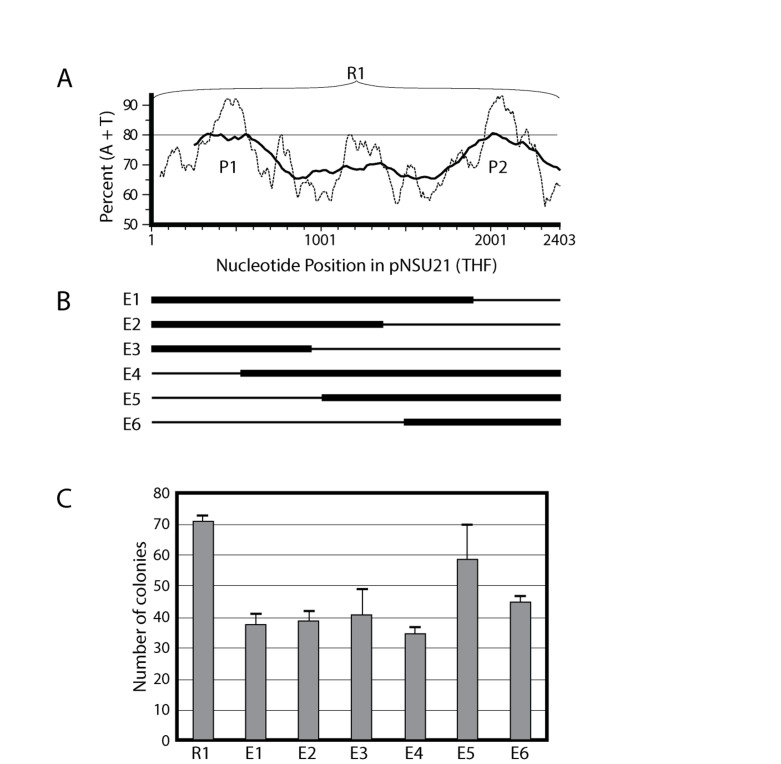
Exonuclease III deletions of Region 1. ( **A**) Intact Region 1 with AT content, expanded from Figure 2. ( **B**) For constructs E1–E6, the thin lines show the portions of R1 that were deleted, and the thick lines show the portions of R1 remaining in each construct. The deletions were constructed using the Promega Erase-A-Base Kit. See Materials and Methods for details. ( **C**) Results of ARS assays for the plasmids containing the deleted versions of Region 1 shown in panel B. Compare with Figure 2D.

In order to better localize the ARS element(s) within R1, we used an exonuclease III deletion procedure to reduce the size of R1 by ~500 bp, ~1000 bp, and ~1.5 kbp from both ends ( Figure 3B; constructs E1–E6). Each of the resulting constructs contained at least one of the peaks of high AT content (P1 and P2) found within R1 ( Figure 3A,B). When we assayed these constructs for ARS activity ( Figure 3C) we found that all of them were active, but none was as active as R1. The results in Figure 3 suggest that either AT peak within R1 is sufficient to serve as an origin of replication. Thus there are two closely spaced, but independent, ARS elements close to the centromere-proximal end of the THF.

### Replication timing of the THF and of R1

To test the replication timing of the THF and of R1 in their plasmid context, we used the hydroxyurea (HU) block-and-release procedure ^8, 27^ to synchronize cells. First we treated the cells with HU for 4 hours. This resulted in depletion of the dNTP pools, causing DNA replication to be blocked in early S phase. Early-firing replication origins can fire in the presence of HU, but the replication forks from these origins stall within a short distance (usually less than 10 kb). When cells are released from the HU block, they resume DNA replication. We took time points after release to follow progress of the cells through S phase. We analyzed the DNA from each time point by two-dimensional (2D) agarose gel electrophoresis to test for the presence of replication intermediates (RIs). We probed our blots with vector sequences, so as not to confuse the plasmid signals with those from the corresponding chromosomal sequences. Under our conditions, the time points with the most intense RI signals corresponded to the most frequent times of plasmid replication within the cell population.

**Figure 4.**
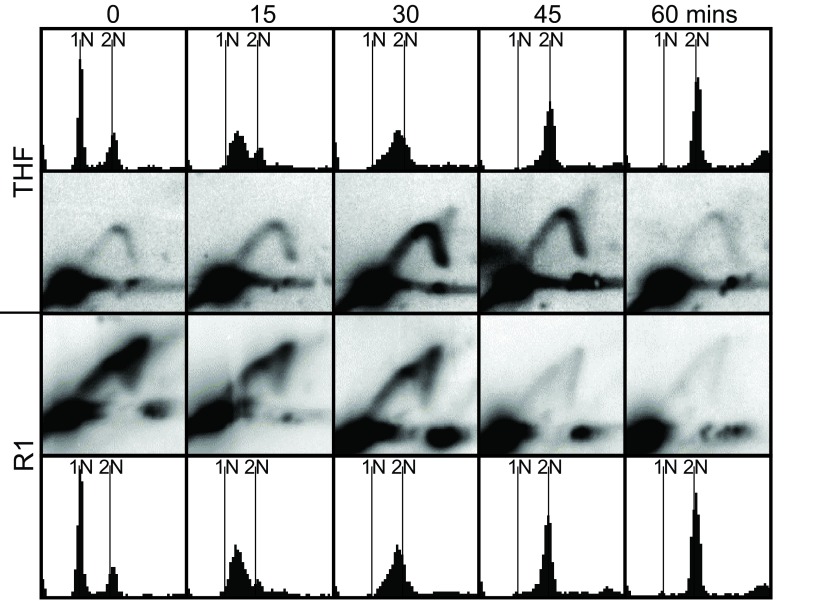
Late replication of the THF; early replication of Region 1. Topmost panels: Flow cytometric analysis of the passage through S phase of cells containing plasmids bearing the intact 8-kb THF. The majority of cells arrested with an approximately 1N DNA content after four-hour treatment with hydroxyurea (HU; 0 minutes). The DNA content of these cells gradually increased after removal of HU (15 minutes through 60 minutes). Upper middle panels: Two-dimensional (2D) gel analyses show that replication intermediates from the plasmid bearing the THF were most abundant 30 and 45 minutes after removal of HU (late S phase). Lower middle panels: Similar 2D gel analyses show that plasmids containing only R1 replicated primarily during the HU block (0 minutes; early S phase) and nearly all replication was complete by 45 minutes. Bottommost panels: Flow cytometry showing progression through S phase of cells containing plasmids bearing only Region 1. These cells progressed through S phase with kinetics indistinguishable from those of cells containing plasmids with the intact THF (topmost panels).

The 2D gel results for the THF-bearing plasmid ( Figure 4, top panels) show that the majority of cells replicated the plasmid at 30 or 45 minutes after release from the HU block. There are RIs visible in all time points, but they are most abundant at 30 minutes and somewhat less abundant at 45 minutes. The corresponding flow cytometry results ( Figure 4, topmost panels) show that these times correspond to late S phase, consistent with the observed late replication of the THF in the chromosome ^8^. The 2D gel results for the R1 subfragment indicate that RIs were most abundant at 0 minutes ( Figure 4, lower panels), meaning that these RIs formed in the presence of HU and were therefore a consequence of early replication. RIs persisted, though less abundant, at 15 and 30 minutes, and then nearly disappeared at 45 and 60 minutes. Thus the majority of R1 plasmid replication took place in early S phase, while most THF plasmid replication took place in late S phase. This leads us to conclude that there are *cis*-acting sequences within the THF that force late replication, but these sequences are not located within R1.

### Attempt to localize the THF sequences that regulate replication timing

**Figure 5.**
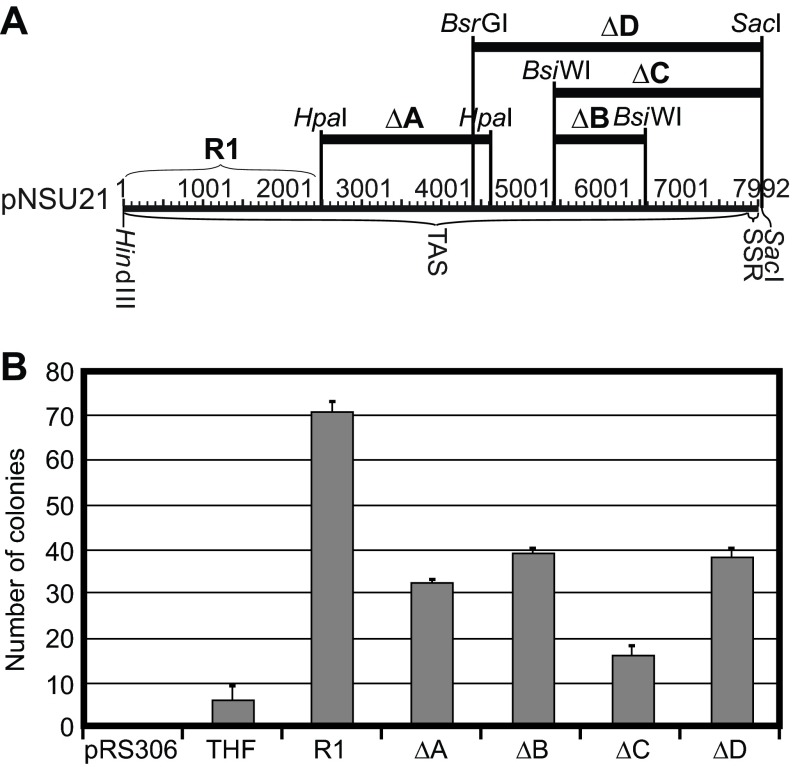
Effects on ARS activity of deleting portions of the leftmost 2/3 of the THF. ( **A**) Diagram of the THF and the regions deleted from it. The thick horizontal bars show regions deleted. The restriction enzyme sites bordering each deletion are indicated. ( **B**) Results of ARS assays for controls and for plasmids containing the deleted THF sequences shown in panel A. Compare with Figure 2D and Figure 3C.

Next we examined the effects of deleting various sequences from the THF, while leaving R1 intact. Segments of the THF bordered by the restriction sites indicated in Figure 5A were deleted from the THF. Each of the deletions left the R1 portion of the THF intact. Figure 5B shows the ARS activities of the four deletion-containing fragments compared to the activities of pRS306 (negative control), the intact THF, and the R1 fragment by itself (see also Figure 2D). Each of the deletions significantly *increased* ARS activity compared to the intact THF, but each also *decreased* activity compared to the R1 fragment by itself. This means that, although each deletion removed some of the sequences that inhibit firing of the origins within the R1 fragment, none of the deletions removed all of the inhibitory sequences. Inhibitory sequences must be distributed broadly throughout the rightmost 70% of the THF.

**Figure 6.**
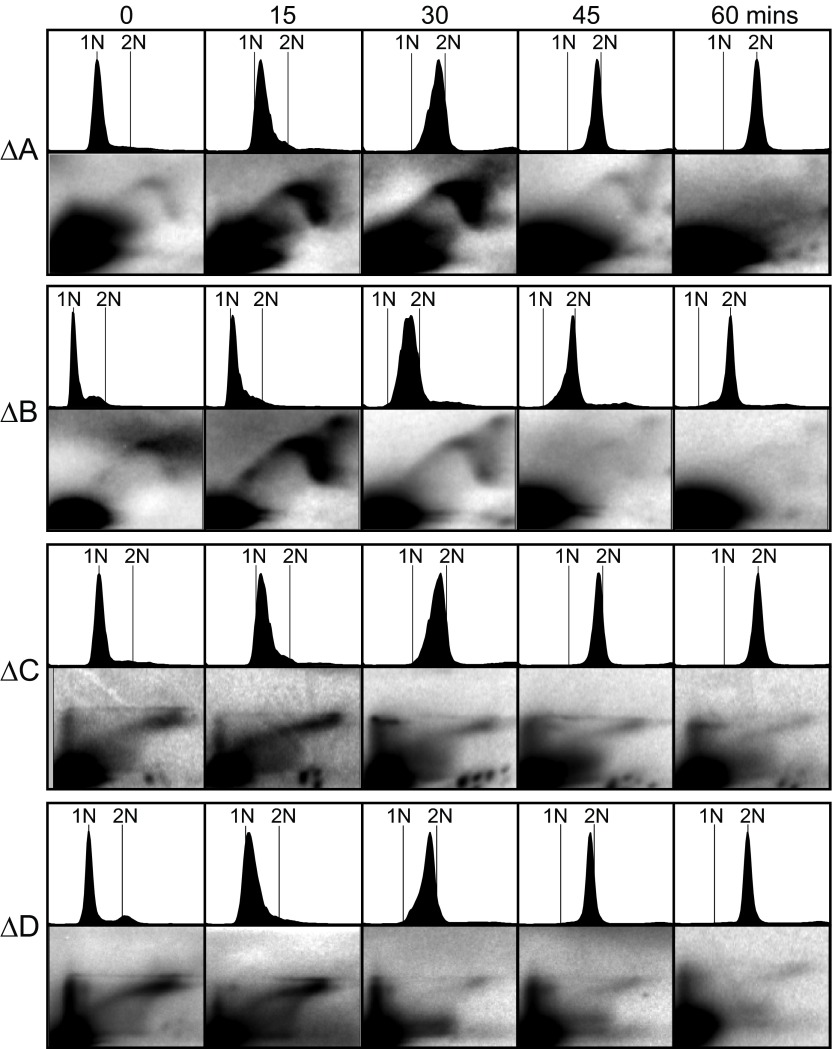
Effects on replication timing of deletions within the rightmost 2/3 of the THF. Flow cytometry and 2D gel results (as in Figure 4) are shown for plasmids containing each of the deletions described in Figure 5. The unusual shapes of the replication-intermediate signals for ∆C and ∆D are a result of using the standard 2D-gel-electrophoresis procedure on large (>6 kb) DNA fragments. For ∆A and ∆B typical Y-arc shapes were generated, because in those cases the large plasmid was digested with two restriction enzymes, and the detected restriction fragment was approximately 4.3 kb.

Next we measured the replication times of these deletion plasmids, using the same methods as in Figure 4. Flow cytometry showed that the rate of passage through S phase was almost identical for each of the four strains containing deleted plasmids ( Figure 6). Each deletion ( Figure 6) accelerated replication timing compared to the intact THF ( Figure 4). Deletion ∆A had the least effect. Each of the other deletions, ∆B, ∆C, and ∆D, accelerated replication timing so much that the strongest RI signal was in early S phase (15-minute time point). The strongest effect was produced by the largest deletion, ∆D. However, none of these deletions ( Figure 6) permitted replication to be as early as the R1 subfragment by itself ( Figure 4), for which the strongest RI signal is at the 0-minute time point. We conclude that *cis*-acting sequences promoting late replication are distributed throughout the rightmost 70% of the THF and are especially abundant in the rightmost 45% covered by deletion ∆D.

**Figure 7.**
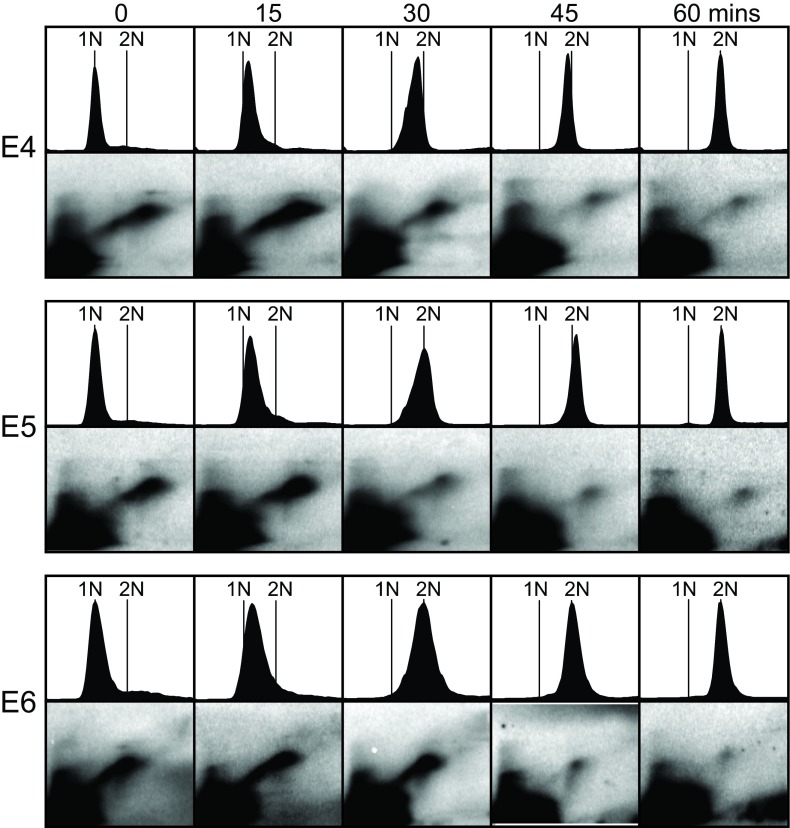
Effects on replication timing of deletions within Region 1. Flow cytometry and 2D gel results (as in Figure 4 and Figure 6) are shown for plasmids containing some of the deletions described in Figure 3B. The shapes of the Y arcs are unusual due to the large sizes of the fragments analyzed.

We also examined the replication timings of the two individual ARS elements within the R1 fragment ( Figure 3). The flow cytometry profiles and 2D results are shown in Figure 7 for deletions E4, E5, and E6 (deletions E1, E2, and E3 produced essentially identical results). Similar to the intact R1 fragment ( Figure 4), these deletions maintained high concentrations of RIs at the 0-, 15- and 30-minute time points. In contrast to the R1 fragment, their RI concentrations may have been slightly greater at 15 minutes than at 0 minutes. The fact that deletions E4 and E5 appear to complete replication somewhat earlier than deletion E6 ( Figure 7) suggests the possibility that *cis*-acting sequences promoting *early* replication may be present in the stretch of about 500 bp that is deleted in E6 but not E4 or E5 ( Figure 3). Thus multiple *cis*-acting sequences within the 8-kb THF regulate its late replication timing, with sequences in the rightmost 45% favoring late replication and sequences in the leftmost portion (R1 fragment) possibly promoting early replication.

### Importance of the checkpoint kinase, Cds1, for late replication of the THF

**Figure 8.**
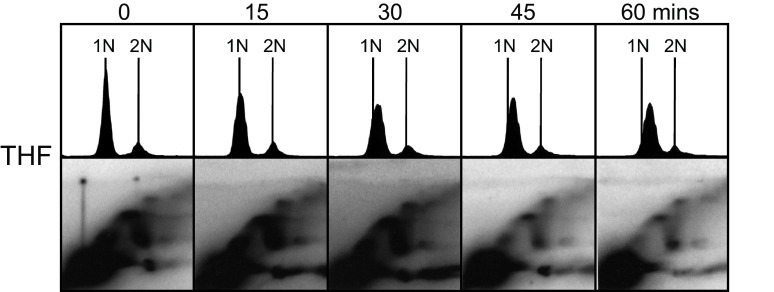
Late replication of the THF requires the Cds1 checkpoint kinase. Flow cytometry and 2D gel results (as in Figure 4, Figure 6 and Figure 7) are shown for the plasmid containing the intact THF, replicating within cells lacking the Cds1 checkpoint kinase. Compare these results with those in Figure 4 (top two panels), where the same plasmid was replicating in cells with functional Cds1 kinase.

In the fission yeast replication checkpoint pathway, the Rad3 kinase phosphorylates and contributes to activation of the downstream checkpoint kinase, Cds1 (reviewed in ^28^). The fact that telomeres are shortened in *rad3*∆ cells suggests that Rad3 is important for telomere structure ^29^. However, deletion of the gene encoding the downstream checkpoint kinase, Cds1, has no detectable effect on telomere structure ^29^. Two laboratories ^14, 15^ have demonstrated that loss of either Rad3 or Cds1 permits earlier replication in HU-treated cells of TAS in their *natural context* near the ends of chromosomes. We wanted to test whether this advanced replication timing in checkpoint-deficient cells would also apply to the 8-kb THF described above ( Figure 1, FIgure 2, Figure 4 and Figure 5) in a *plasmid context*. Figure 8 shows the results of an HU block-and-release experiment on *cds1*Δ cells bearing this plasmid: the THF-containing plasmid replicated primarily in early S phase, in contrast to its late replication when in wild-type cells ( Figure 4). Comparison between wild-type cells ( Figure 4) and *cds1*∆ cells ( Figure 8) is somewhat complicated by the fact that, after release from the HU block, passage through the rest of S phase is much slower in *cds1*∆ cells than in wild-type cells, as previously demonstrated by Kim and Huberman ^8^ (S.M. Kim and J.A. Huberman, unpublished). Despite this complication, the 2D gel results ( Figure 8) show maximum RI abundance at 0–30 minutes, corresponding to early S phase. The abundance of RIs decreased as the cells progressed further into S phase (45 and 60 minutes). This indicates a loss of late-replication-timing control for the plasmid containing the 8-kb THF in *cds1*Δ cells, similar to the loss of late-replication-timing control for TAS in checkpoint-deficient cells in their chromosomal context ^14, 15^.

## Discussion

### Two closely-spaced replication origins near telomeres at chromosome ends

We have identified two DNA replication origins in the fission yeast telomere-associated sequences, TAS. The origins were found within a cloned, sequenced 8-kb Telomeric *Hin*dIII Fragment (THF) originally identified by Sugawara and cloned by him into the plasmid, pMLC12 ( Figure 1; ^1^). We recloned the intact THF and eight portions of it, regions R1–R8, into a yeast shuttle plasmid (pRS306 ^18^) and then tested these plasmids for origin activity. We found that all of the origin activity was confined to region 1, which in its chromosomal context is 6–8 kb from the simple-sequence repeats at chromosome ends ( Figure 2).

In fission yeast, replication origins tend to be highly AT-rich. Conversely, high AT richness is a good predictor of origin locations and activities ^22, 23^. Within region R1 there are two peaks of high (>80%) AT content, P1 and P2 ( Figure 2, Figure 3). Measurements of the origin activities of exonuclease-generated fragments of region R1 showed that fragments containing P1 alone or P2 alone were individually capable of functioning as replication origins, although neither P1 nor P2 was as active as region R1, which contained both P1 and P2 ( Figure 3). Thus region R1 contains not one, but two, active replication origins, and these two origins are nearly adjacent to each other, separated by about 1.6 kb. The two origins are closer to telomeres than are any other origins within the fission yeast genome ( Figure 1, Figure 2).

Interestingly, there are two additional high-AT peaks, P3 and P4, within the THF ( Figure 2). Each of regions R2, R5 and R6 contains at least one of peaks P3 and P4. Region R5 contains P2 and P3. Although peak P2 is active by itself ( Figure 3), and although P3 and P4 are just as AT-rich as P1 and P2, none of regions R2, R5 or R6 has detectable origin activity ( Figure 2). This suggests that regions R2, R5 and R6 must contain *cis*-acting sequences that inhibit origin function, and region R1 must be relatively free of such sequences. Our preliminary investigation revealed ( Figure 5) that these inhibitory sequences may well be located in multiple places within regions R2, R5 and R6, and additional inhibitory sequences also appear to be present in regions R3, R4 and R7.

### Multiple *cis*-acting sequences cooperatively restrict replication of the THF to late S phase

In its chromosomal context, the THF replicates in late S phase ^8^. Interestingly, we found that the intact THF also replicates late in a plasmid context ( Figure 4). This suggests that the late replication of telomere-proximal sequences in fission yeast chromosomes may not be a consequence of their proximity to chromosome ends, since the plasmids we constructed were circular, not linear.

Instead, our results suggest that *cis*-acting sequences within the non-R1 portion of the THF must be responsible for the late replication of the THF. A plasmid bearing only region R1 replicated early in S phase, while a plasmid containing the complete 8-kb THF replicated late ( Figure 4). None of four deletions, which together covered all of the non-R1 portion of the THF, was sufficient to permit the remaining fragment to replicate as early as does region R1 by itself ( Figure 4, Figure 5, Figure 6). These observations suggest that *cis*-acting sequences promoting late replication are widely distributed throughout the non-R1 portion of the THF.

**Figure 9.**
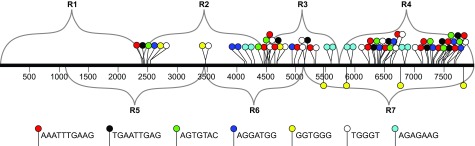
The identities and locations of some sequence motifs present in regions R2–R7, but not in R1. The diagram is an expansion of Figure 2B, with the positions of color-coded sequence motifs added. The motifs were identified using Meme software (see Materials and Methods).

What could these late-replication-determining *cis*-acting sequences be? We don’t know the answer, but a non-exhaustive search for sequence motifs present only in the non-R1 portion of the THF revealed multiple possibilities ( Figure 9). In addition to, or instead of, contributing to late replication of the THF, the motifs identified in Figure 9 that are located in regions R2, R5 and R6 may also contribute to suppression of origin activity in these regions by the AT-rich peaks P3 and P4 ( Figure 2).

It is interesting that two of the motifs in Figure 9, GGTGGG and TGGGT, seem related to the *cis*-acting "LCS" motif (GKKGGGGGAW, where K represents G or T and W represents A or T) previously identified by Yompakdee and Huberman as being capable of restraining plasmids containing *ars727* or *ars3001* to replication in late S phase ^25^. All three of these G- and T-rich motifs are also related to a motif (CNWWGTGGGGG, where N represents any nucleotide and W represents A or T) observed by Hayano *et al.* to be enriched in sites that bind Rif1, a protein essential for normal replication timing in fission yeast ^30^.

These similarities suggest that the Rif1 protein may be at least partially responsible for late replication of the THF.

Another sequence motif that should be considered a candidate for contributing to low origin efficiency and/or to late replication timing is the simple-sequence telomeric repeat motif (GGTTA-CA), 12 copies of which are located between coordinates 7882 and 7981of the pNSU21 sequence shown in Figure 2 and Figure 5. If this motif plays a role in reducing replication efficiency or promoting late replication timing, it is likely that it does so only as a repeat sequence, because a single copy is found in region R1. Since a plasmid containing only R1 replicates efficiently in early S phase, the single copy of GGTTACA within R1 is clearly insufficient to reduce replication efficiency or retard its timing. Even if the 12 repeats of GGTTACA contribute to regulating plasmid replication efficiency or timing, they cannot be the only motif doing so, because plasmids lacking these repeats (∆C and ∆D, Figure 5, Figure 6) nevertheless replicate less efficiently and later than a plasmid containing only region R1 ( Figure 4, Figure 5, Figure 6).

### Checkpoint regulation of telomere-proximal replication origins in a plasmid context

We noted above that a plasmid containing the intact, approximately 8-kb telomeric *Hin*dIII fragment (THF) replicates in late S phase, at the same time as the corresponding chromosomal sequences. Late replication of the *chromosomal* sequences requires the Cds1 checkpoint kinase ^8^. Similarly, in this study we found that late replication of the THF-containing *circular plasmid* also requires the Cds1 kinase ( Figure 8). We conclude that, at least for the approximately 8-kb stretch of telomere-associated sequences (TAS) at the ends of chromosomes 1 and 2, both checkpoint-dependence of late replication and late replication itself are dependent primarily on *cis*-acting sequences, not on proximity to the physical end of a linear chromosome.

## Materials and methods

### Strains and growth conditions

We used three *Schizosaccharomyces pombe* strains, *ura4*- *D18* h ^-^^31^, 501 *ura4* ^-^ h ^-^^32^ and *cds1*:: *ura4 ura4-D18 leu1-32 ade6-704* h ^-^^33^. Cells were cultured in YES medium (5 g/l yeast extract, 30 g/l glucose, 225 mg/l adenine, uracil, histidine, lysine hydrochloride, and leucine; ^34^) at 30°C. Cells were also grown on Edinburgh minimal medium + supplements (ade-nine, histidine, lysine hydrochloride, and leucine) (EMMS-ura; ^34^) at 30°C for 8 days for transformation with plasmids. *E. coli* (DH5α) cells (New England Biolabs) were used to clone TAS sequences prior to yeast transformation.

### Yeast transformation procedure (ARS assay)

*ura4*- *D18* h ^-^ fission yeast cells ^31^ were transformed by a modified version of the lithium acetate procedure as described ^35^. For each sample, two transformations – one with 500 ng and the other with 1000 ng plasmid DNA – were carried out simultaneously. The results shown are an average of 3 separate experiments, with triplicate plating of 1% of each transformation mixture. The cells were plated on EMMS-ura medium and incubated at 30°C. Colonies were counted on day 8 after transformation.

### Nucleotide sequences

*S. pombe* telomere sequences were downloaded from ftp://ftp.sanger.ac.uk//pub2/yeast/sequences/pombe/telomeres. The *S. pombe* reference genome of October, 2008, was downloaded from ftp://ftp.sanger.ac.uk//pub2/yeast/pombe/Chromosome_contigs/OLD/20080922.

### The 8-kb Telomeric *Hin*dIII Fragment (THF)

Neal Sugawara cloned and sequenced multiple fission yeast telomeric restriction fragments ^1^. We obtained his 8-kb clone, pNSU21, from the Sanger Center (see http://www.pombase.org/tools/clone-and-mapping-resources; send e-mail to archives@sanger.ac.uk).

### Cloning

The plasmid, pRS306 ^18^, was used as recipient for all clonings, due to its *URA3* selectable marker and convenient multiple cloning site. The 8-kb *Hin*dIII- *Sac*I fragment (the THF) from pNSU21 was cloned between the *Hin*dIII and *Sac*I sites of pRS306.

Fragments representing Regions 1–8 were generated by PCR amplification from the 8-kb THF. The primers are listed in Table S1. Due to multiple internal sequence repetitions within the TAS fragment, all of the PCR reactions resulted in multiple bands. The band of interest (identified by its electrophoretic mobility) was gel-purified and cloned into pRS306. Many of the primers had restriction enzyme sequences added for cloning purposes.

### Exonuclease III deletions

As implied in Table S1, the PCR fragment representing Region R1 was cloned into pRS306 between the *Hin*dIII and *Bam*H1 sites. The Promega Erase-a-Base Kit, which employs exonuclease III, was used to make the deletions indicated in Figure 3. For deletions E1–E3, the plasmid containing R1 was first digested with *Bam*HI and *Kpn*I. The *Kpn*I site is protected from exonuclease III digestion, so deletions were made leftward (orientation of Figure 3) from the *Bam*HI site. For deletions E4–E6, the plasmid containing R1 was digested with *Hin*dIII and *Kpn*I. In this case, the deletions were made rightward from the *Hin*dIII site. After exonuclease III digestions, the plasmids were recircularized. Plasmids containing deletions of the desired size were first selected by gel electrophoretic size characterization and then confirmed by sequencing.

### Cell synchronization by hydroxyurea block and release

Cells were grown in EMMS-ura medium to maintain plasmid selection. On the day of the experiment, they were transferred to YES at a density of 0.7 to 1.2 × 10 ^7^ cells/ml at 30°C and treated with 25 mM hydroxyurea (Sigma-Aldrich) for 4 hours. The cells were then washed twice with 30°C water by 5-min centrifugations in a Sorvall RC5B or RC6 using the GS3 or SLA-3000 rotors respectively. The cells were then resuspended in 30°C YES, and 200-mL samples were collected at T0 (release) and every 15 minutes thereafter for 1 hour. Sodium azide (Sigma-Aldrich) was added to 0.01% to block further cellular metabolism. The cell samples were pelleted in a Sorvall RC5B GSA rotor and stored at -70°C until needed.

### Flow cytometry

An aliquot of cells was fixed in 70% ethanol and stored at 4°C. The day before flow analysis, the fixed cells were washed twice in 50 mM sodium citrate, then resuspended in 500 µL of 50 mM sodium citrate with 100 µg/ml RNAse A (Sigma-Aldrich). These cells were incubated overnight at 37°C. The next day, 500 µL of 50 mM sodium citrate supplemented with 2 µM Sytox Green (Molecular Probes) were added to the cells. The cells were sonicated and stored at 4°C until analyzed by flow cytometry on a Becton Dickinson FACScan.

### DNA preparation

200 mL of cells (7–10 × 10 ^6^ cells/mL) were used to prepare DNA for neutral-neutral 2D gel electrophoresis by previously described methods from Huberman *et al.* ^36^ and Brewer and Fangman ^37^. The procedure can be found at our website: http://joelhuberman.net/HubermanLabArchives/2D_Gel_Docs_HTML.html. The BND-cellulose enrichment of replication intermediates was omitted from the procedure. The DNA was digested by the enzymes used in cloning the TAS into pRS306. The final southern blots were hybridized with vector sequences alone ( *Sac*1-linearized pRS306 DNA), so as not to confuse the results for the plasmids with those for chromosomal TAS. The autoradiograms were scanned using a Molecular Dynamics PhosphorImager to produce 16-bit TIFF files. IP Lab 3.5 software (originally from Scanalytics.com, currently updated and sold as iVision-Mac by BioVision Technologies) was used to adjust image intensities so that the intensities of the 1N spots would be uniform for each set of figures.

### Motif identification

We used MEME software ( http://meme.sdsc.edu/meme/cgi-bin/meme.cgi) to identify sequence motifs present in regions R2–R7 but not in R1. We used regions R2, R3 and R4 as positive sequences and region R1 as a negative sequence. We looked for zero or one occurrence of each motif among the three positive sequences, with maximum motif width 10 bases and minimum width 5 bases. We set the number of different motifs at 20, the minimum number of sites at 2, and the maximum number of sites at 50. From the many motifs identified, we selected those with the broadest distributions and highest frequencies through R2–R4.
