# Supplementary material for: Identification of two telomere-proximal fission yeast DNA replication origins constrained by nearby cis-acting sequences to replicate in late S phase
Source: F1000Res. 2012 Dec 4;1:58. [Version 1] doi: 10.12688/f1000research.1-58.v1 (PMC3790605; doi:10.12688/f1000research.1-58.v1)
Supplement: Peer review history

# Peer review: Referee response for version 1

**Published:** 2012-12-17

**Authors:** Jurg Bahler

**Affiliations:** 1 Department of Genetics, Evolution & Environment and UCL Cancer Institute, University College London, London, UK

I have read this submission. I believe that I have an appropriate level of expertise to confirm that it is of an acceptable scientific standard.

# Peer review: Referee response for version 1

**Published:** 2012-12-14

**Authors:** Toru M Nakamura

**Affiliations:** 1 Department of Biochemistry and Molecular Genetics, University of Illinois at Chicago, Chicago, USA

The current manuscript identifies new replication origins near telomeres in fission yeast. The information provided will serve as a useful starting point for future investigations.

The most interesting finding is the identification of potential cis-acting sequences within subtelomeres that can cause telomeric ARS to be late replicating. As suggested by the authors, it would be very interesting to determine if those cis-acting regulatory sequences might collaborate with Rif1 to enforce late S-phase replication of telomeres in the future.

Overall, the manuscript is clearly written and conclusions are supported by experiments shown. On the other hand, 2D gel data, especially Figures 6 & 7, are somewhat hard to understand since the signal for replication intermediates is weak and there appears to be non-specific background signals from hybridization on those gels as well. It may be helpful to provide cartoons of 2D gel hybridization signals to clearly indicate what part of the gel corresponds to replication intermediate signals.

I have read this submission. I believe that I have an appropriate level of expertise to confirm that it is of an acceptable scientific standard.

# Response

**Published:** 2012-12-19

**Authors:** Joel Huberman

**Affiliations:** Department of Molecular and Cellular Biology, Roswell Park Cancer Institute , USA

We thank Dr. Nakamura for his useful comments. Especially helpful is his suggestion that cartoons of 2D gel hybridization signals might help clarify the unusual gel patterns in Figures 6 and 7. We will create such a cartoon and submit it as a supplementary figure in the appropriately revised version of the manuscript.

# Peer review: Referee response for version 1

**Published:** 2012-12-07

**Authors:** Hisao Masai

**Affiliations:** 1 Department of Genome Medicine, Tokyo Metropolitan Institute of Medical Science, Tokyo, Japan

This manuscript provides useful information regarding the mechanisms of how telomere-proximal origins are suppressed for initiation in early S phase.

The authors identified two origins near the telomere and found that they are suppressed by cis-acting elements present in adjacent segments (closer to the telomere). These latter sequences enforce late replication or suppression of replication even on the circular plasmid bearing the ARS fragment containing the above two putative origins. The late replication depend on the Cds1 function, suggesting that checkpoint suppresses the replication of from these origins through the putative cis-acting “late replication regulatory sequences”.

The data provided are not complete to dissect the sequences and cellular functions required for late replication of subtelomere segments, but provide useful data that can be explored for further studies.

Some of the 2D data are not so clear (Figures 6 and 7) and could be redone under modified conditions that would give better separation of RIs. The analyses of ∆A, ∆B, ∆C and ∆D are a bit confusing. Although it is stated that ∆A had least effect and still is replicated late (Figure 6), ∆A is comparable to ∆B and ∆D and better than ∆C in ARS assays in Figure 5. A potential role of rif1 in the late replication is suggested in Discussion, and it is certainly of interest to see the replication timing of THF in rif1∆ cells.

Figure 5 title; “leftmost 2/3″ should be “rightmost 2/3″.

I have read this submission. I believe that I have an appropriate level of expertise to confirm that it is of an acceptable scientific standard.

# Response

**Published:** 2012-12-07

**Authors:** Joel Huberman

**Affiliations:** Department of Molecular and Cellular Biology, Roswell Park Cancer Institute , USA

We thank Dr. Masai for his useful comments and for pointing out our careless mistake in the title for Figure 5.

We agree that some of the 2D data in Figures 6 and 7 are not optimally clear, because the analyzed fragments were large and therefore migrated anomalously. Despite that problem, however, the times at which signal strengths from replication intermediates were maximal are obvious in Figures 6 and 7, so we believe that our conclusions are sound. Unfortunately, our laboratory is now closed, due to my retirement and to Dr. Chaudari’s move to Houston, so we cannot redo the experiments with smaller fragment sizes.

We also agree that it would be highly interesting to test the effect of the rif1 deletion on replication extents and timings of the THF and its subfragments. We hope that other laboratories will also be curious about these possibilities and will follow up with appropriate experiments. As implied in our manuscript and as pointed out by Dr. Masai, we think that further investigations of the replication properties of the THF and its subfragments (and also of mutant THFs and subfragments in which various sequence motifs have been modified) in appropriate mutant strains will shed much more light on the mechanisms regulating both extent and timing of replication of these two telomere-proximal origins.
